# Time Course of Corticospinal Excitability and Autonomic Function Interplay during and Following Monopolar tDCS

**DOI:** 10.3389/fpsyt.2014.00086

**Published:** 2014-07-21

**Authors:** Emiliano Santarnecchi, Matteo Feurra, Federico Barneschi, Maurizio Acampa, Giovanni Bianco, David Cioncoloni, Alessandro Rossi, Simone Rossi

**Affiliations:** ^1^Unit of Neurology and Neurophysiology, Department of Medicine, Surgery and Neuroscience, University of Siena, Siena, Italy; ^2^Brain Investigation and Neuromodulation Lab, University of Siena, Siena, Italy; ^3^U.O.C. Stroke Unit, Department of Medicine, Surgery and Neuroscience, Le Scotte Policlinic, Siena, Italy

**Keywords:** transcranial direct current stimulation, transcranial magnetic stimulation, safety, neuromodulation, vital parameters

## Abstract

While polarity-specific after-effects of monopolar transcranial direct current stimulation (tDCS) on corticospinal excitability are well-documented, modulation of vital parameters due to current spread through the brainstem is still a matter of debate, raising potential concerns about its use through the general public, as well as for neurorehabilitation purposes. We monitored online and after-effects of monopolar tDCS (primary motor cortex) in 10 healthy subjects by adopting a neuronavigated transcranial magnetic stimulation (TMS)/tDCS combined protocol. Motor evoked potentials (MEPs) together with vital parameters [e.g., blood pressure, heart-rate variability (HRV), and sympathovagal balance] were recorded and monitored before, during, and after anodal, cathodal, or sham tDCS. Ten MEPs, every 2.5-min time windows, were recorded from the right first dorsal interosseous (FDI), while 5-min epochs were used to record vital parameters. The protocol included 15 min of pre-tDCS and of online tDCS (anodal, cathodal, or sham). After-effects were recorded for 30 min. We showed a polarity-independent stabilization of cortical excitability level, a polarity-specific after-effect for cathodal and anodal stimulation, and an absence of persistent excitability changes during online stimulation. No significant effects on vital parameters emerged both during and after tDCS, while a linear increase in systolic/diastolic blood pressure and HRV was observed during each tDCS condition, as a possible unspecific response to experimental demands. Taken together, current findings provide new insights on the safety of monopolar tDCS, promoting its application both in research and clinical settings.

## Introduction

Non-invasive brain stimulation (NIBS) techniques are increasingly used as potential treatments for numerous neurological and psychiatric conditions (1–5). The rationale behind the therapeutic use of such techniques is that both repetitive transcranial magnetic stimulation (rTMS) and transcranial direct current stimulation (tDCS) may produce changes in the cortical excitability of the stimulated neural networks, outlasting the stimulation period. While rTMS mainly induces long-lasting changes in synaptic efficacy (6), tDCS changes cortical excitability in a polarity-specific manner by modifying the intracellular ion concentrations in the cortical tissue, through an action at the level of the membrane potential: cathodal tDCS (C-tDCS) induces inhibition of the stimulated network, while anodal stimulation (A-tDCS) acts in an opposite way (1–4, 7, 8). Additional action mechanisms of tDCS such as changes in synaptic strength (9) or changes in the resting activity of glial cells (10) have been also documented. tDCS-induced changes in cortical excitability may have positive behavioral consequences, if the dysfunction of the NIBS-conditioned network is associated with the generation/maintenance of a given symptom. Besides having an important role in investigating the physiology of motor (7, 11–14) and visual areas (15–17), where changes of cortical excitability can be directly indexed by neurophysiological parameters, tDCS research has also shown to have a strong translational power, with promising scenarios concerning new treatment options for neurological and psychiatric disorders. Moreover, tDCS devices are freely available on the web market for unsupervised home usage as neuroenhancers (18), opening a worrisome scenario by a medical and social perspective (18, 19).

Specifically, tDCS can be delivered by adopting bipolar (4, 7) or monopolar (20, 21) montages: the former implies an “active” (either cathode or anode) and a “reference” electrode placed on the scalp surface, while the latter uses a “reference” placed on an extracephalic target (shoulder, leg, arm, etc.). In this case, the induced electric field may flow toward brainstem structures, thereby potentially affecting the function of the neural centers, which regulate autonomic nervous system functions (22). However, the effects of tDCS techniques on vital parameters as blood pressure, heart-rate variability (HRV), sympathetic/parasympathetic balance, and respiration frequency, are still controversial (23). While a potential modulation of sympathetic activity via the stimulation of motor cortex (24), dorsolateral prefrontal cortex (DLPFC) (25), as well parietal (26), occipital (27), and temporal (28) lobes have been already demonstrated, the variability in terms of electrode montages, study design (cross-sectional vs. parallel), blinding, and tDCS modality applied across studies posit the need for further investigations (19). Furthermore, whether tDCS exerts its effect over the autonomic system mostly during or right after its delivery is still a matter of debate (29), as well as the reliability of tridimensional head models of local current field as vehicle to investigate the aforementioned issues (5). Finally, it is noteworthy that the identification of potential effect of tDCS over CNS structures that govern autonomic nervous function may candidate several pathological conditions as potential targets for treatments, like arterial hypertension (30), vasovagal syncope (31), obesity (29), diabetes (32), and migraine (33), while holding a drawback in terms of its application in neurological and psychiatric population in which such secondary effects could represent a limit instead.

Therefore, to originally investigate the online and after-effects of monopolar tDCS on autonomic functions, we simultaneously acquired corticospinal excitability levels and vital parameter data before, during, and after tDCS using a combined TMS–tDCS set-up. Differently from previous investigations available to date, this approach allowed us to originally investigate the effect of tDCS on vital parameters in light of a net measure of its effect on cortical excitability. By monitoring such dynamics through the entire experiment, we will be able to describe the possible modulation of vital parameters as a response to the fluctuations in cortical excitability induced by tDCS.

## Materials and Methods

### Participants

Ten tDCS-naïve and fully right-handed healthy volunteers with normal neurological examinations took part in the study (five female; mean age 26 ± 3 years). The experiment was performed with the approval of the Ethical Committee of Siena University. An informed consent was obtained from all subjects according to the Declaration of Helsinki. Following a cross-over design, all participants blindly underwent three separate sessions of randomized A-tDCS, C-tDCS, and sham tDCS (S-tDCS) of the dominant primary motor cortex (left M1), each spaced about 1 week apart (5–7 days). They sat comfortably in a reclining chair with their arm fully relaxed in a natural position and their hands pronated on a pillow.

### Electrophysiological and vital parameter recordings

**Figure 1:**
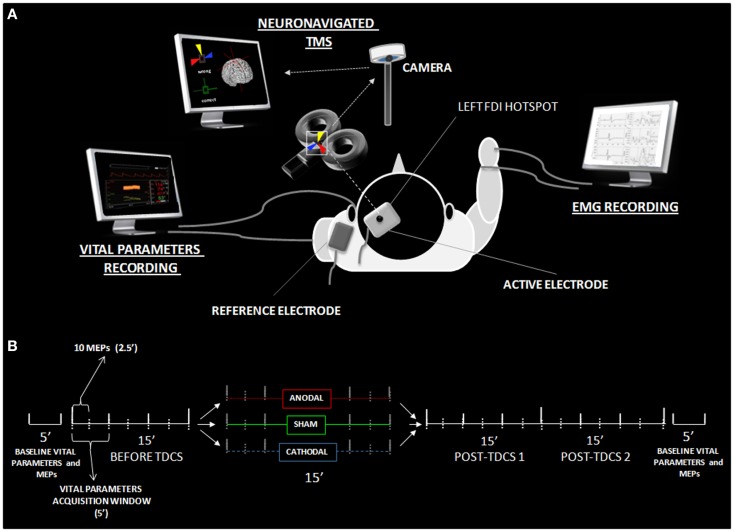
**Experimental details**. **(A)** Shows the experimental set-up with active-reference electrode positionings, the neuronavigated TMS coil placement over the active electrode, and settings for the EMG and vital parameter recordings. **(B)** Reports the time course of data acquisition, illustrating the different time windows utilized in the statistical analysis. MEPs and vital parameters were acquired, respectively, every 2.5 and 5 min. Analysis was performed on both high-resolution data and 15 min collapsed windows (before, during, tDCS, post 1 and post 2). Additional vital parameter and MEP data were collected 5 min before and after the experiment, in order to obtain basal-level values for data normalization.

Each recording session started with the identification of the left M1 by searching for the hotspot of the contralateral first dorsal interosseous (FDI) muscle, according to standard single-pulse focal coil TMS session parameters (34). The active tDCS electrode was then applied to the left M1. Then, electrodes for the cardiovascular parameters recordings were applied (35). The TMS hotspot was checked again in order to ensure a stable set-up immediately before the experiment began. The whole time course of the experiment is displayed in Figure 1. In order to guarantee the gold-standard set-up for minimization of trial-to-trial variability of cortical excitability, we used a TMS neuronavigation system throughout the entire experiment, which is *per se* an original approach into the investigation of tDCS-induced changes in cortical excitability.

### TMS protocol, MEP recordings, and neuronavigation procedures

Single-pulse TMS was delivered via a monophasic Bistim 200 stimulator (MagStim) with an approximately 2.2 T maximal output connected to a standard figure-of-eight shaped coil (diameter of each wing 70 mm). The coil was held tangential to the scalp, with the handle pointing backward and laterally, angled at 45° from the midline sagittal axis of each participant’s head. The “hotspot” was marked with a pencil at the scalp location that triggered motor evoked potentials (MEPs) of >50 μV with 50% probability, corresponding to the resting motor threshold (10). A neuronavigation system (SofTaxic by EMS srl) was used throughout the experiment in order to allow for the exact repositioning of the TMS coil within and across experimental sessions (36). This procedure provided three-dimensional online information regarding the initial and actual coil placements, while minimizing the variability of TMS-induced electric fields directly measured within a scalp model (37). As in previous original transcranial electrical stimulation studies combined with online TMS (15), the tDCS target electrode was placed on the TMS hotspot. Such a procedure allowed for the collection of MEPs online during the delivery of tDCS. In order to overcome the electrode resistance due to the additional thickness under the coil, the TMS intensity was set at 120–130% of the individual motor threshold to elicit fairly stable basal MEPs of 600–800 μV in the right FDI. Ag–AgCl adhesive electrodes were positioned over the target muscle in a belly-tendon bipolar montage, with the active electrode placed over the muscle belly of the target muscle and the reference electrode on the nearest finger joint. MEPs were recorded using a four-channel electromyography (Phasis, EBNeuro), with a bandpass filter of 20 Hz–2 kHz, sampled at 20 kHz, with a gain range of 0.1–1.5 mV. A total time epoch of 200 ms was analyzed for each trial, with the first 100 ms serving as a pre-trigger analysis period, in order to monitor and exclude those trials that might be contaminated by unwanted background electromyographic activity. Ten MEPs with the same onset latency for each time window (2.5 min; see Figure 1) were collected. This number of trials/condition was adopted in order to keep the experimental time shorter, to avoid tiring the subjects. The onset of every TMS pulse was jittered in a randomized fashion ranging from 10 to 20 s from the previous one.

### Vital parameters acquisition

Heart-rate variability, blood pressure variability (BPV), and spontaneous baroreflex sensitivity (BRS) were measured in all participants before, during, and after anodal, cathodal, and S-tDCS (see Figure 1). The monitoring lasted a total of 60 min, including 15 min before stimulation, 15 min of tDCS stimulation, and 30 min after. Recordings were made in a quiet room with the ambient temperature between 20 and 24°C, during spontaneous breathing (i.e., stable 17–20 respiratory acts/min). Standard electrocardiographic and hemodynamic parameters were monitored and recorded by means of the Task Force Monitor 3040 apparatus (CNSystems, Graz, Austria).

#### Heart-rate variability and blood pressure variability

Analog-to-digital conversion of ECG recordings was performed at 1000 Hz. Photoplethysmographic signals of finger systolic (sBP), diastolic (dBP), and mean blood pressure (mBP) were collected on a beat-to-beat basis, by using a vascular unloading technique (16) and calibrated with the data obtained from intermittent automated oscillometric blood pressure measurements on the contralateral arm (brachial artery). The algorithm used for the short-term HRV analysis was an autoregressive method (16th-order model) (38); total power and three main frequency bands were measured: very low (VLF; <0.03 Hz), low (LF; 0.03–0.15 Hz), and high (HF; 0.15–0.5 Hz) frequency components. The ratio LF/HF was calculated as an expression of the sympathovagal balance (39). The LF component is correlated with peripheral vasomotor activity and may be under sympathetic and parasympathetic influence, whereas parasympathetic activity is considered as the major contributor to HF power. The measurement of VLF, LF, HF, and total power was made in absolute values of power (square milliseconds). LF and HF components are presented also in normalized units (n.u.), obtained as follows: HFn.u. = [HF square milliseconds/(LF square milliseconds + HF square milliseconds) × 100] (35). Separate power spectra were constructed for diastolic blood pressure (dBP), calculating three frequency domains: total power (0.03–0.5 Hz), low (LF) (0.03–0.15 Hz), and high (HF) frequency components (0.15–0.50 Hz). The dBP spectrum power had units of square millimeter of mercury. LF of BPV was associated with sympathetic nervous activity and HF of BPV was associated with the mechanical effect of respiration. LF-dBP/HF-dBP ratio was calculated as an index of sympathovagal balance. As heart-rate is influenced by respiratory drive, the ratio of the LF component of dBP and HF component of heart-rate (LF-dBP/HF-HR) was also calculated.

#### Transcranial direct current stimulation

A monopolar tDCS montage was applied, using one active electrode (sponge/rubber electrode, 35 cm^2^) placed over the left M1 and a reference electrode placed on the left shoulder. Standard saline solution (NaCl 9%) was used to soak the electrode sponges. Impedances were kept below 10 kOhm throughout all stimulation sessions. tDCS was applied through an Eldith^®^ DC-stimulator (neuroConn GmbH, Ilmenau, Germany) using the following parameters: anodal/cathodal stimulation = 1 mA; fade in/out 8 s; sham stimulation = 1 mA, fade in/out 8 s, 45 s on; average current density at the stimulation electrode = ~28.5 μA/cm^2^. In order to attenuate anxiety and/or possible side effects induced by the novel experience of transcranial stimulation, a brief 30 s stimulation was applied on each participant as training (1 mA, fade in 8 s, 46 s on) before the acquisition of vital parameters and tDCS. Participants were also familiarized with the TMS during the identification of the motor threshold.

### Experimental design

In order to obtain a stable baseline evaluation of cortical excitability to be used for reference in the statistical analysis, 10 MEPs were acquired before each experimental session (baseline MEPs hereafter). For the same reason, vital parameters were recorded for 5 min before (baseline-VP) and after the experiment (baseline-VP-2). The order of the experimental sessions (A-tDCS, C-tDCS, and S-tDCS) was counterbalanced between subjects and spaced 5–7 days apart each. Once the experiment started, 15 min of pre-tDCS, 15 min of online tDCS, and 30 min of post-tDCS (split into two time windows of 15 min, post-tDCS1 and post-tDCS2) were run. Each 15-min epoch was subdivided into windows of 2.5 min (Figure 1). Acquisition of 10 MEPs was performed every 2.5 min. The experiment lasted 60 min, including 24 time windows of MEP acquisition (Figure 1). In order to get reliable data, vital parameters were recorded and averaged using 5 min windows (21).

### Data analysis

#### Motor evoked potentials

Peak-to-peak maximal amplitude of each MEP was calculated offline; 10 artifact-free FDI MEPs/conditions (spaced-out by a ~10″ interval) were averaged for each subject. MEP size was normalized as the percentage of the collapsed peak-to-peak amplitude for baseline MEPs (10). Analyses were performed on both data at the maximum time resolution (10 MEPs every 2.5 min, roughly corresponding to a continuous cortico-excitability evaluation) and on MEP amplitudes averaged within the four 15-min long experimental blocks (pre-tDCS, online tDCS, post-tDCS1, and post-tDCS2).

#### Time windows analysis

**Figure 2:**
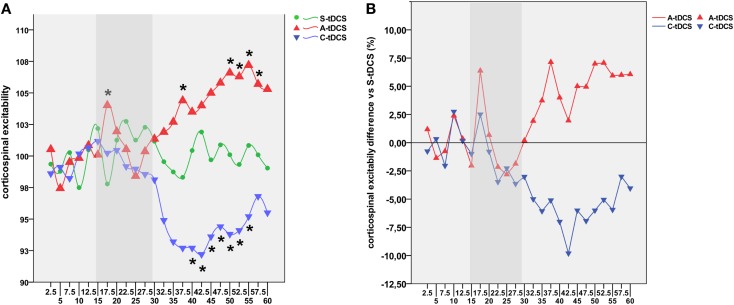
**Time course of tDCS effects**. **(A)** Shows the high-resolution time course of MEP values for sham (green line), anodal (red line), and cathodal (blue line) tDCS conditions. Time points define different experimental conditions, namely pre-tDCS (2.5–15′), online tDCS (17.5–30′, gray band), post-tDCS1 (32.5–45′), and post-tDCS2 (47.5–60′). The *y*-axis refers to the corticospinal excitability values normalized using baseline MEPs acquired 5 min before the experiment for each condition. Each time point represents the average value of eight consecutive MEPs acquired within the 2.5 s wide window. Asterisks indicate time points showing a significant difference with respect to S-tDCS (*p* < 0.05). **(B)** Represents the average percentage of increase or decrease in cortical excitability for A-tDCS and C-tDCS respect with S-tDCS (straight line).

The statistical analysis was primarily run on a finer time scale of 2.5 min latency intervals to assess fluctuations of MEP amplitudes under tDCS application throughout the whole experimental session. Therefore, a three-way repeated measures (RM) ANOVA with a three-level factor “type of stimulation” (anodal, cathodal, and sham), six-level factor “time window” (2.5, 5, 7.5, 10, 12.5, 15), and three-level factor “stimulation block” (online tDCS, post-tDCS1, and post-tDCS2) was run in order to check for any cortical excitability change during the experimental sessions, which included 15 min of online tDCS and 30 min of post-tDCS divided and referred as post-tDCS1 (15 min) and post-tDCS2 (15 min) (Figure 2). Huynh–Feldt correction was applied when necessary to compensate for violating the sphericity assumption. The order of tDCS conditions was included as a covariate in the model. In the presence of significant interactions, corrected pairwise comparisons were performed using a Bonferroni correction. We set *p* < 0.05 as the criterion for statistical significance.

#### tDCS blocks analysis

In order to better highlight the global effects of online and offline tDCS, data were collapsed into three 15-min blocks according to the experimental condition (online tDCS, post-tDCS1, and post-tDCS2). Briefly, MEP data were normalized by reducing the heteroscedasticity between different conditions, thereby allowing for more reliable inter-individual comparisons. Each individual value was log normalized (natural logarithm) (40) and then normalized as the percentage of the baseline condition, which included the average of the collapsed peak-to-peak amplitude means collected during every pre-tDCS time window. Thus, an RM-ANOVA with a three-level factor “type of stimulation” (anodal, cathodal, and sham) and a three-level factor “collapsed time windows” was run. As in previous analysis, Huynh–Feldt correction was applied when necessary to compensate for the violation of the sphericity assumption. The order of tDCS conditions was again included as a covariate. Given the dimensionality reduction performed by collapsing the data into three main time windows in the presence of significant interactions, the Bonferroni correction for pairwise comparisons was applied in order to limit the experiment-wise error rate to α and to maximize the power of the test detecting pairwise differences. We again set *p* < 0.05 as the criterion for statistical significance.

#### Vital parameters

Thirteen vital parameters (systolic [sBP], diastolic [dBP] and mean blood pressure [mBP]; heart-rate [HR]; very low [VLF-RRI], low [LF-RRI] and high [HF-RRI] frequency components of HRV; vagal tone of RRI in normalized unit [HF nu-RRI]; sympathetic tone of RRI in normalized unit [LF nu-RRI]; sympathovagal balance [LF/HF]; sympathovagal balance of RRI [LF/HF-RRI]; power spectral density of RRI [PSD-RRI]; R–R interval [RRI]) were included for the analysis. Details about data acquisition and indexes computation are included as supplemental material. To assess fluctuations of each vital parameter throughout the experiment, every session was divided into 5-min intervals to get enough data for a reliable parameter estimation (35). Data from each variable measured during the tDCS protocols were normalized as the percentage change with respect to baseline-VP values. A two-way RM-ANOVA with a 3-level factor “type of stimulation” (anodal, cathodal, and sham) and 11-level factor “time window” (5, 10, 15, 20, 25, 30, 35, 40, 45, 50, 55, and 60) was run on each vital parameter in order to check for any change during all the experimental sessions (online tDCS, post-tDCS1, and post-tDCS2). The order of tDCS conditions was again included as a covariate.

#### Interplay between cortical excitability and sympathovagal balance

In order to capture a statistical dependency between cortical excitability and vital parameters, which could have not potentially reach statistical significance in the ANCOVA, the long-term concurrent, online–offline, MEP-vital parameters acquisition design we adopted allowed us to documented the correlation coefficient between cortical excitability and sympathovagal balance parameters during both A-tDCS and C-tDCS (see Figures S1, S2, and S3 in Supplementary Material for an overview of their temporal dynamics during the experiment). Additionally, apart from spare evidences (29), little is known about the relationship between spontaneous oscillations in brain excitability and autonomic function during spontaneous rest, i.e., when no external perturbation are delivered. By using the data acquired during S-tDCS, we also looked for the presence of physiological correlations occurring during spontaneous resting-state, assuming the existence of a significant relationship between cortical excitability and sympathovagal balance.

In order to run the analysis, MEP data were converted to a 5-min time scale by averaging pairs of consecutive 2.5 min time windows, after which a partial correlation analysis was performed between MEP and vital parameters for each tDCS condition (*p* < 0.05 Bonferroni corrected; controlling for age and gender). Vital parameters showing a significant correlation have been included in separate linear regression analyses, including MEP amplitude as an independent variable, vital parameters as a dependent variable, and gender and age as covariates.

## Results

### Motor evoked potentials

#### Time windows

The time window RM-ANOVA showed a main factor effect of “type of stimulation” [*F*_(2; 18)_ = 3.883, MSE = 49095.33, *p* = 0.04]. *Post hoc* comparisons showed a trend toward significance in results between anodal and cathodal stimulation effects (*p* = 0.098). A significant three-way interaction between “type of stimulation,” “time window,” and “stimulation block” emerged [*F*_(16.35; 147.23)_ = 1.021, MSE = 1895.45, *p* = 0.014].

*Post hoc* comparisons showed a robust increase of corticospinal excitability by online A-tDCS vs. S-tDCS (*p* = 0.004) for the initial 2.5 min of online stimulation, while corticospinal excitability did not change during C-tDCS. During the 30 min after the stimulator had been switched off, A-tDCS and C-tDCS showed different temporal patterns of modulation over time: C-tDCS significantly impacted cortical excitability mostly during post-tDCS1, while A-tDCS effects reached significance only during post-tDCS2 (Figure 2). Additionally, as expected, the directionality of A-tDCS and C-tDCS effect was different: C-tDCS exerted an inhibition of corticospinal excitability with respect to S-tDCS at 10 min (*p* = 0.022), 12.5 min (*p* = 0.014), and 15 min (*p* = 0.019) of post-tDCS1, as well as at 2.5 min (*p* = 0.019), 5 min (*p* = 0.021), 10 min (*p* = 0.020) and 12.5 min (*p* = 0.026) of post-tDCS2. Differently, A-tDCS increased corticospinal excitability at 7.5 min (*p* = 0.022) of post-tDCS1, and at 5 min (*p* = 0.018), 7.5 min (*p* = 0.009), 10 min (*p* = 0.011), and 12.5 min (*p* = 0.010) of post-tDCS2.

Several significant differences between anodal and C-tDCS also emerged during the entire post-tDCS1 and post-tDCS2 time windows, starting from 5 min of post-tDCS1 onward (see Figure 2). Additionally, as shown in Figure S1 in Supplementary Material, the exogenous electric field – irrespective of its polarity – did interact with ongoing corticospinal excitability by regularizing MEP time series autocorrelations. Specifically, autocorrelation (range +1/−1) defines the degree of similarity between a given time series and its lagged version over successive time intervals, allowing one to observe non-random behavior, such as patterns over time. Thus, autocorrelation might be considered an index of “persistence,” that is, the tendency for the system’s time series to remain in the same state from one observation to the next. Observed results highlight an increased self-predictive power of excitability levels during anodal and C-tDCS with respect to sham condition, which is an expression of stimulation influence over physiological excitability fluctuation at rest.

#### tDCS blocks

Repeated measures-ANOVA on collapsed data showed a main factor effect of “type of stimulation” [*F*_(2; 18)_ = 4, MSE = 176.97, *p* = 0.037]. *Post hoc* comparisons showed an inhibition of C-tDCS with respect to A-tDCS (*p* = 0.008) and close to significance with respect to sham (*p* = 0.074), irrespective of online/offline tDCS conditions.

**Figure 3:**
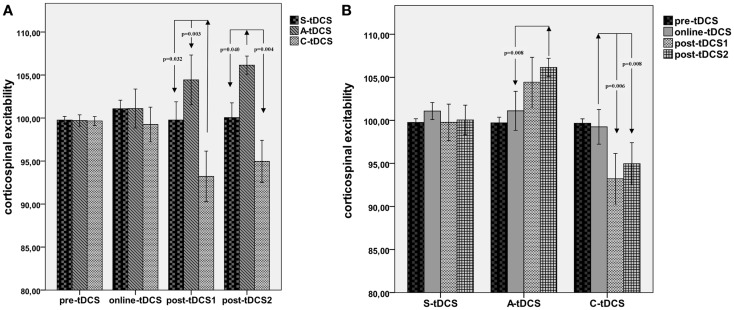
**Collapsed time windows analysis**. **(A)** Shows corticospinal excitability values collapsed in the 15 min before, 15 min during, 15 min post-1 and 30 min post-2 each tDCS condition. A repeated measures ANOVA highlighted significant differences between C-tDCS and S-tDCS/A-tDCS during the first 15 min after the stimulator had been switched off, while A-tDCS induced an increase in the average MEP level during the second 15 min blocks (post-tDCS2) with respect to S-tDCS and C-tDCS. **(B)** Reports tDCS condition-wide corticospinal excitability values showing a significant difference between post-tDCS2 compared to online tDCS for A-tDCS, as well for post-tDCS1 and 2 with respect to online tDCS during C-tDCS. *P* values refer to Bonferroni corrected pairwise comparisons.

A significant “collapsed time window” × “type of stimulation” effect emerged [*F*_(2; 19)_ = 7.43, MSE = 21.34, *p* = 0.012]. As reported in Figure 3, *post hoc* comparisons showed that C-tDCS robustly inhibited corticospinal excitability with respect to A-tDCS (*p* = 0.003) and S-tDCS (*p* = 0.032) during post-tDCS1, while A-tDCS increased corticospinal excitability with respect to cathodal (*p* = 0.004) and sham (*p* = 0.040) during post-tDCS2. Moreover, A-tDCS showed a significant difference between MEP values collected during online tDCS and post-tDCS2 conditions (*p* = 0.008), while C-tDCS showed significantly smaller MEPs in the post-tDCS1 (*p* = 0.006) and post-tDCS2 (*p* = 0.006) with respect to online tDCS.

#### Vital parameters

The vital parameters ANOVA with a three-level factor “type of stimulation” (anodal, cathodal, and sham) and a 12-level factor “time window” (5, 10, 15, 20, 25, 30, 35, 40, 45, 50, 55, 60) showed a significant main effect for “time window” referring to sBP [*F*_(5.067; 40.53)_ = 2.566, MSE = 314.175, *p* = 0.041], dPB [*F*_(5.433; 39.51)_ = 3.121, MSE = 298.346, *p* = 0.032], and LFnu-RRI [*F*_(4.45; 35.67)_ = 3.242, MSE = 1254.21, *p* = 0.019] values, driven by their increase during online tDCS and post-tDCS2. No significant effects for “type of stimulation” and “type of stimulation × time window” interactions were observed (Table 1). A graphical representation of cortical excitability and autonomic functions fluctuations through the entire experiment is included in Figures S2, S3, and S4 in Supplementary Material.

**Table 1:** **Analysis of vital parameters changes during tDCS**.

Vital parameters	Main effect *type of stimulation*	Main effect *time window*	Interaction *type of stimulation x time window*
RRI	*F*_(2; 16)_ = 0.624, MSE = 160.75, *p* = 0.548	*F*_(4.25; 34.06)_ = 1.015, MSE = 37.26, *p* = 0.416	*F*_(8.11; 64.89)_ = 0.469, MSE = 15.25, *p* = 0.876
HR	*F*_(2; 16)_ = 0.660, MSE = 180.01, *p* = 0.529	*F*_(5.15; 46.38)_ = 0.758, MSE = 26.11, *p* = 0.588	*F*_(7.81; 70.32)_ = 0.685, MSE = 28.60, *p* = 0.700
dBP	*F*_(2; 16)_ = 0.843, MSE = 5767.40, *p* = 0.449	*F*_(5.433; 39.51)_ = 3.121, MSE = 298.346.03, *p* = ***0.032***	*F*_(6.97; 55.82)_ = 0.948, MSE = 181.32, *p* = 0.478
sBP	*F*_(2; 16)_ = 0.288, MSE = 178.15, *p* = 0.753	*F*_(5.067; 40.53)_ = 2.566, MSE = 314.175, *p* = ***0.041***	*F*_(3.48; 27.83)_ = 1.064, MSE = 418.72 *p* = 0.387
mBP	*F*_(2; 16)_ = 1.648, MSE = 55490791.72, *p* = 0.224	*F*_(1.00; 8.00)_ = 1.664, MSE = 613274933.89, *p* = 0.233	*F*_(1.00; 8.00)_ = 1.663, MSE = 1225436492.59, *p* = 0.233
HFnu-RRI	*F*_(2; 16)_ = 0.430, MSE = 5086.52, *p* = 0.658	*F*_(5.74; 45.94)_ =*2.264, MSE =* *3520.45, p* *=* *0.056*	*F*_(8.41; 67.32)_ = 0.955, MSE = 2203.57, *p* = 0.481
HF-RRI	*F*_(2; 16)_ = 0.741, MSE = 192618.06, *p* = 0.490	*F*_(4.34; 39.06)_ = 0.677, MSE = 70002.75, *p* = 0.624	*F*_(4.43; 39.92)_ = 1.529, MSE = 289323.31, *p* = 0.208
LF-HF	*F*_(2; 16)_ = 0.200, MSE = 2034.99, *p* = 0.821	*F*_(2.72; 21.83)_ = 1.276, MSE = 2034.99, *p* = 0.306	*F*_(3.26; 26.10)_ = 1.630, MSE = 50667.10, *p* = 0.204
LFnu-RRI	*F*_(2; 16)_ = 0.778, MSE = 1163.60, *p* = 0.475	*F*_(4.45; 35.67)_ = 3.242, MSE = 1254.21, *p* = ***0.019***	*F*_(4.91; 39.33)_ = 0.567, MSE = 50667.10, *p* = 0.722
LF-RRI	*F*_(2; 16)_ = 0.780, MSE = 11653.84, *p* = 0.475	*F*_(4.95; 39.64)_ = 0.836, MSE = 9725.48, *p* = 0.531	*F*_(7.32; 58.57)_ = 1.170, MSE = 14644.94, *p* = 0.334
PSD-RRI	*F*_(2; 16)_ = 1.167, MSE = 182205250502, *p* = 0.336	*F*_(11; 88)_ = 1.397, MSE = 216945059569.7, *p* = 0.188	*F*_(11; 176)_ = 1.474, MSE = 228499618417.4, *p* = 0.088
VLF-RRI	*F*_(2; 16)_ = 1.035, MSE = 273407362256.3, *p* = 0.378	*F*_(1.68; 13.49)_ = 1.637, MSE = 2808481572147, *p* = 0.231	*F*_(1.69; 13.57)_ = 1.637, MSE = 6264609602110, *p* = 0.198
LF/HF-RRI	*F*_(2; 16)_ = 0.200, MSE = 2034.991893701, *p* = 0.821	*F*_(2.72; 21.83)_ = 1.276, MSE = 24565.72764047, *p* = 0.306	*F*_(3.26; 26.10)_ = 1.630, MSE = 50667.10187743, *p* = 0.204

*Statistics for the main effects and interactions regarding the modulation of vital parameters during tDCS conditions are reported here. In bold, significant ANCOVA results (*p* < 0.05, Bonferroni corrected) are highlighted, while results trending to significance are reported in italics*.

#### Interplay between cortical excitability and sympathovagal balance

**Figure 4:**
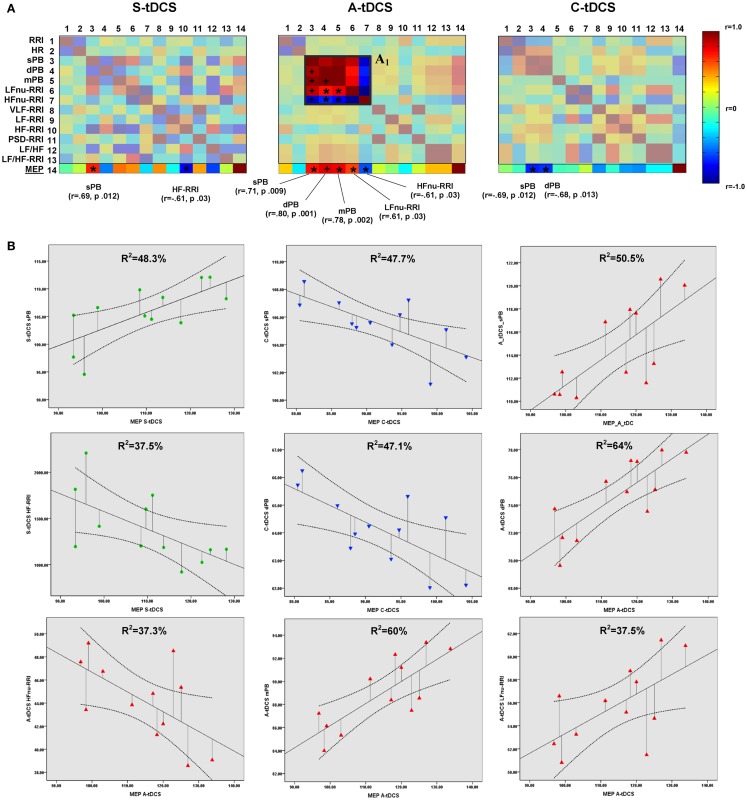
**Correlations between corticospinal excitability and vital parameters**. **(A)** Shows the results of partial correlation analyses between vital parameters and MEP amplitudes computed by properly converting the latter to a 5-min time window resolution. The last matrix rows refers solely to motor evoked potential correlation coefficients (color-coded according to the right side bar) for different tDCS conditions, with Pearson’s *r* coefficients and *P* values reported for sympathovagal parameters, showing a significant correlation. Insert A_1_ highlights the increased positive correlation between systolic (sBP), diastolic (dBP), mean blood pressure (mBP), and LFnu-RRI values during anodal stimulation, and the negative correlation between these values and HFnu-RRI (*p < 0.05; ^+^*p* < 0.01). Significant values entered a further linear regression analysis, whose results are shown in **(B)**. A linear fit line with confidence intervals and the amount of variance in vital parameters explained by MEP values (*R*^2^) are reported.

Partial correlation analyses showed significant correlations between MEP amplitudes and vital parameters for each tDCS condition (Figure 4A). Linear regression analyses confirmed the predictive power of corticospinal excitability over vital parameters: S-tDCS → sPB (*t* = 3.056, β =0.695, *R*^2^ = 48.3%, *p* = 0.012); S-tDCS → HF-RRI (*t* = −2.451, β = −0.613, *R*^2^ = 37.5%, *p* = 0.034); A-tDCS → dPB (*t* = 4.213, β = 0.800, *R*^2^ = 64%, *p* = 0.002); A-tDCS → sPB (*t* = 3.193, β = 0.711, *R*^2^ = 50.5%, *p* = 0.010); A- tDCS → mPB (*t* = 3.921, β = −0.778, *R*^2^ = 60%, *p* = 0.003); A-tDCS → LFnu-RRI (*t* = −2.438, β = −0.611, *R*^2^ = 37.5%, *p* = 0.035); A-tDCS → HFnu-RRI (*t* = −2.438, β = −0.611, *R*^2^ = 37.3%, *p* = 0.035); C-tDCS → sPB (*t* = −3.020, β = −0.691, *R*^2^ = 47.7%, *p* = 0.013); C-tDCS → dPB (*t* = −2.981, β = −0.686, *R*^2^ = 47.1%, *p* = 0.014) (see Figure 4B). Apart from anodal and C-tDCS, results obtained during S-tDCS highlighted a spontaneous interplay between excitability level and cardiac output.

## Discussion

The present study was designed to explore monopolar tDCS safety issues related to the eventual spread of current toward deep brainstem structures. We originally adopted a cross-over, single blind design aimed at evaluating both online and offline effects of tDCS over autonomic nervous functions, while concurrently recording corticospinal excitability. We applied anodal, cathodal, and S-tDCS over left primary motor cortex and recorded both neuronavigated MEPs (in order to reduce the variability of TMS electric fields induced in the brain) (37) and vital parameters before, during, and after tDCS delivery. Results confirmed previously reported polarity-specific tDCS after-effects on corticospinal excitability of similar size but slightly shifted in time for cathodal and A-tDCS, while also providing the first evidence of no significant changes in cortical excitability (MEP size) during online stimulation, if care is taken to stabilize MEPs’ size by using a neuronavigation system.

In respect to vital parameters, a linear increase in systolic/dBP and HRV was observed in each tDCS condition during the experiment, as a possible expression of a slight discomfort due to the experimental set-up and its length. No other significant changes in autonomic functioning have been detected. Current data suggest how this particular monopolar tDCS set-up does not induce significant adverse effects concerning heart-rate and BPV, both during and after the stimulation, while inducing appreciable effects over cortical excitability, thus making it a safe tool for NIBS.

Regarding any kind of therapeutic intervention, safety is a primary issue (“primum non-nocere,” as said by Latin physicians). While the safety of rTMS applications in healthy subjects and patient populations has been recently revised and updated by an international panel of experts (41), there is only meta-analytical evidence reporting on adverse effects and the safety of tDCS (42, 43), with the most common findings described as minor effects, such as transient itching, tingling, headache, burning sensation, and discomfort. Understanding the dynamics and potential weaknesses of electrical stimulation is evidently critical in order to determine and control consequent behavioral, and eventually clinical, outcomes (19), also considering the current exponential diffusion that such technique is going through even within the general public (29).

Transcranial electrical stimulation safety might be strongly related to the electrode placement (14, 44). The large majority of tDCS literature involves a bipolar montage, with both active and reference electrodes placed on the scalp (7, 15, 45). This might lead to undesired excitability changes also under the reference electrode, an issue that can be ruled out by increasing the size of the reference electrode, pointing at a reduction of local current density (9), or through the usage of extracephalic references placed on the mastoids (46, 47), arms (11, 45), or legs (48). Evidence about the current flow between the scalp and extracephalic electrode suggests that there may be stimulation of deep brain stem regions (46). Monopolar montages opened new theoretical issues about possible adverse effects and at the same time, the possibility to use NIBS as a tool to influence sympathetic outflow and, eventually, blood pressure, thus providing a novel therapeutic tool for human arterial hypertension (11). A recent contribution by Moliadze (44) suggested how there might exist a between-electrodes distance impact on tDCS after-effects, with longer distances leading to weaker effects in terms of duration and magnitude, and, consequently, to the need of an *ad hoc* adjustment of stimulation intensities. On the contrary, it seems that there is no difference in terms of stimulation efficacy by using ipsi/contralateral reference electrodes or by targeting dominant or non-dominant hemisphere (49).

Our results support the safety of monopolar tDCS, highlighting that neither global “stimulation” effects nor specific “type of stimulation” alterations in vital parameters occurred during or after tDCS delivery. However, we also documented that, aside from its effect over corticospinal excitability, A-tDCS does seem to induce an increased synchronization between blood pressure and HRV values, referring to sympathetic activity. Considering the role of the sympathetic nervous system in blood pressure regulation, as well as evidence about a sympathetic nervous activation as responsible for blood pressure elevation in essential hypertension (50), such a finding possibly explains previous evidence promoting tDCS as an arterial hypertension modulator (22).

Clearly, aforementioned findings concerning the safety of tDCS can be interpreted as reflection of the quantitative effect of tDCS itself over cortical excitability. Polarity-specific after-effects of tDCS delivered over cortical regions are well-established phenomena, described both in their neurophysiological and behavioral manifestations by several authors (45, 51, 52). They depend both on stimulation intensity and duration (51), and their time course resembles a homeostatic response of the conditioned region (8, 39). Generally, experimental data suggest that a weak, moderately long (~minutes) anodal tDCS induces long lasting facilitatory effects, whereas C-tDCS causes inhibitory modulation of cortical excitability (17, 53).

Whether tDCS exerts online effects is a more controversial question. Previous studies suggested that anodal tDCS decreased (10) or increased the MEP amplitude (51) during its application on the motor cortex, while C-tDCS reduced corticospinal excitability if tested online (51). Differential online effects of anodal and cathodal stimulation have been already suggested in terms of intracortical inhibition or facilitation changes (50) with no online effects of A-tDCS, whereas C-tDCS reduced facilitation during, and additionally increased inhibition after its administration. In this study, we observed that A-tDCS increased corticospinal excitability during the initial 2.5–5 min of stimulation. Even if such a transient “peak” of increased excitability (Figure 2) could be ascribed to participants’ rising alertness immediately after stimulator was turned on, this was not detected for either the C-tDCS or the S-tDCS conditions, thus suggesting that A-tDCS may also induce a transient neuromodulatory effect through membrane potential shifts during current delivery.

As far as after-effects are concerned, we observed two different time courses of brain responsiveness for anodal and cathodal stimulation in terms of timing. Even if the magnitude of the observed effect, that is the effect size of significant comparisons, is small to medium (5–7.5% for A-tDCS, almost 10% for C-tDCS), main results suggest a stronger and wider inhibitory effect for cathodal stimulation, recognizable as an immediate drop of corticospinal excitability right after tDCS offset and lasting for almost 20 min (Figure 2). Differently, A-tDCS resulted in a less accentuated but constant increase of excitability, which reached its maximum after 15-min from tDCS offset (Figure 3). These results are in line with evidence from the literature, suggesting weaker effects for A-tDCS respect to cathodal (9). Altogether, these and our findings would indicate that the inhibitory after-effect of C-tDCS is definitely more robust than the facilitatory one induced by anodal tDCS, even though we did not find a remarkable difference in the magnitude of modulation. It is worth noting that these results have been obtained using a neuronavigation system for TMS, a missed methodological approach in previous studies.

A special remark must be drawn for unspecific corticospinal excitability stabilization induced by active tDCS itself (Figure 3). While during and after sham stimulation corticospinal excitability is variable, both anodal and cathodal stimulation induced a “regularization” of the time course of cortical excitability as indexed by the variability of MEP amplitudes (Figure 3). Of interest, Hartwig and colleagues (54) demonstrated that active tDCS may work as a preconditioner in rTMS protocols, with the possibility to “orientate” the effect of a 1 Hz rTMS stimulation toward inhibition or facilitation by simply preceding motor cortex rTMS delivery with cathodal and A-tDCS. Additionally, they argued that these effects might rely on the polarization, and consequently stabilization of postsynaptic activity like that described by the Bienenstock–Cooper–Munro (BCM) rule of synaptic modification.

Finally, our data also support a possible interplay between central nervous system dynamics and peripheral symphatovagal parameters, as shown by the significant correlations between corticospinal excitability, systolic blood pressure (sBP), and short-term HRV values (low-frequency component at 0.03–0.15 Hz) during S-tDCS. Considering that the LF component is an expression of peripheral vasomotor activity and thus under sympathetic and parasympathetic influence, this might also explain the polarity-specific modulation of corticospinal vital parameters as observed during A-tDCS and C-tDCS, which may be, respectively, driven by a sympathetic and parasympathetic response. Interestingly, this would also fit with the significant increase of blood pressure and HRV values correlation induced by A-tDCS. Such possible interpretation strengthens the need to deepen the understanding of the interplay between brain spontaneous excitability levels and vital parameters, while discouraging the role of extracephalic tDCS as a possible modulator of peripheral symphatovagal parameters.

## Conclusion

Current findings provide new insights on the time course of tDCS effects, both on cortical excitability and safety parameters. Using a monopolar montage and a neuronavigated TMS system to monitor cortical excitability changes, the results showed that a robust inhibition of cortical excitability, lasting for at least 30 min after tDCS offset, can be induced by C-DCS, while the excitatory effect of A-tDCS seems slightly less effective and delayed in time. Most importantly, an analysis of vital parameters fluctuations throughout the entire experiment did not show any significant difference across tDCS conditions, both during and after tDCS. These findings highlight the effectiveness and safety of monopolar electrode montage for tDCS application in experimental and clinical settings.

## Conflict of Interest Statement

The authors declare that the research was conducted in the absence of any commercial or financial relationships that could be construed as a potential conflict of interest.

## Supplementary Material

The Supplementary Material for this article can be found online at http://www.frontiersin.org/Journal/10.3389/fpsyt.2014.00086/abstract
