# Supplementary material for: Time Course of Corticospinal Excitability and Autonomic Function Interplay during and Following Monopolar tDCS
Source: Front Psychiatry. 2014 Jul 21;5:86. doi: 10.3389/fpsyt.2014.00086 (PMC4104833; doi:10.3389/fpsyt.2014.00086)
Supplement: Supplementary file 1 [file Presentation1.ZIP › Supp Fig legends.docx]

**Figure S1. MEP data autocorrelation.** Corticospinal excitability autocorrelation analysis highlighted a pattern of MEP amplitude regularization during active tDCS, with an increased self-predictive power and a switch in correlation polarity during DC delivery (grey band; lag = time points -1).

**Figure S2 Time course of cortical excitability (MEPs) and sympathovagal balance**. Each row reports the fluctuation of MEP values and RRI, HR, sBP, dBP, mBP during each experimental condition (S-tDCS, A-tDCS and C-tDCS). In order to allow a visual comparison, MEP values were converted to a 5-minute time resolution and values were reported as z-scores. Time points define different experimental conditions, namely pre-tDCS (1 - 3 = 2.5' - 15’), online-tDCS (4 – 6 = 17.5' - 30’, grey band), post-tDCS1 (7 – 9 = 32.5' – 45’) and post-tDCS2 (10 – 12 = 47.5' – 60’).

**Figure S3. Time course of cortical excitability (MEPs) and sympathovagal balance**. Each row reports the fluctuation of MEP values and LF-nuRRI, HF-nuRRI, VLF-RRI, LF-RRI, during each experimental condition (S-tDCS, A-tDCS and C-tDCS). In order to allow a visual comparison, MEP values were converted to a 5-minute time resolution and values were reported as z-scores. Time points define different experimental conditions, namely pre-tDCS (1 - 3 = 2.5' - 15’), online-tDCS (4 – 6 = 17.5' - 30’, grey band), post-tDCS1 (7 – 9 = 32.5' – 45’) and post-tDCS2 (10 – 12 = 47.5' – 60’).

**Figure S4. Time course of cortical excitability (MEPs) and sympathovagal balance**. Each row reports the fluctuation of MEP values and HF-nuRRI, PSD-nuRRI, LF/HF-RRI, LF/HF, during each experimental condition (S-tDCS, A-tDCS and C-tDCS). In order to allow a visual comparison, MEP values were converted to a 5-minute time resolution and values were reported as z-scores. Time points define different experimental conditions, namely pre-tDCS (1 - 3 = 2.5' - 15’), online-tDCS (4 – 6 = 17.5' - 30’, grey band), post-tDCS1 (7 – 9 = 32.5' – 45’) and post-tDCS2 (10 – 12 = 47.5' – 60’).
