# Supplementary figures and images for: Time Course of Corticospinal Excitability and Autonomic Function Interplay during and Following Monopolar tDCS
Source: Front Psychiatry. 2014 Jul 21;5:86. doi: 10.3389/fpsyt.2014.00086 (PMC4104833; doi:10.3389/fpsyt.2014.00086)

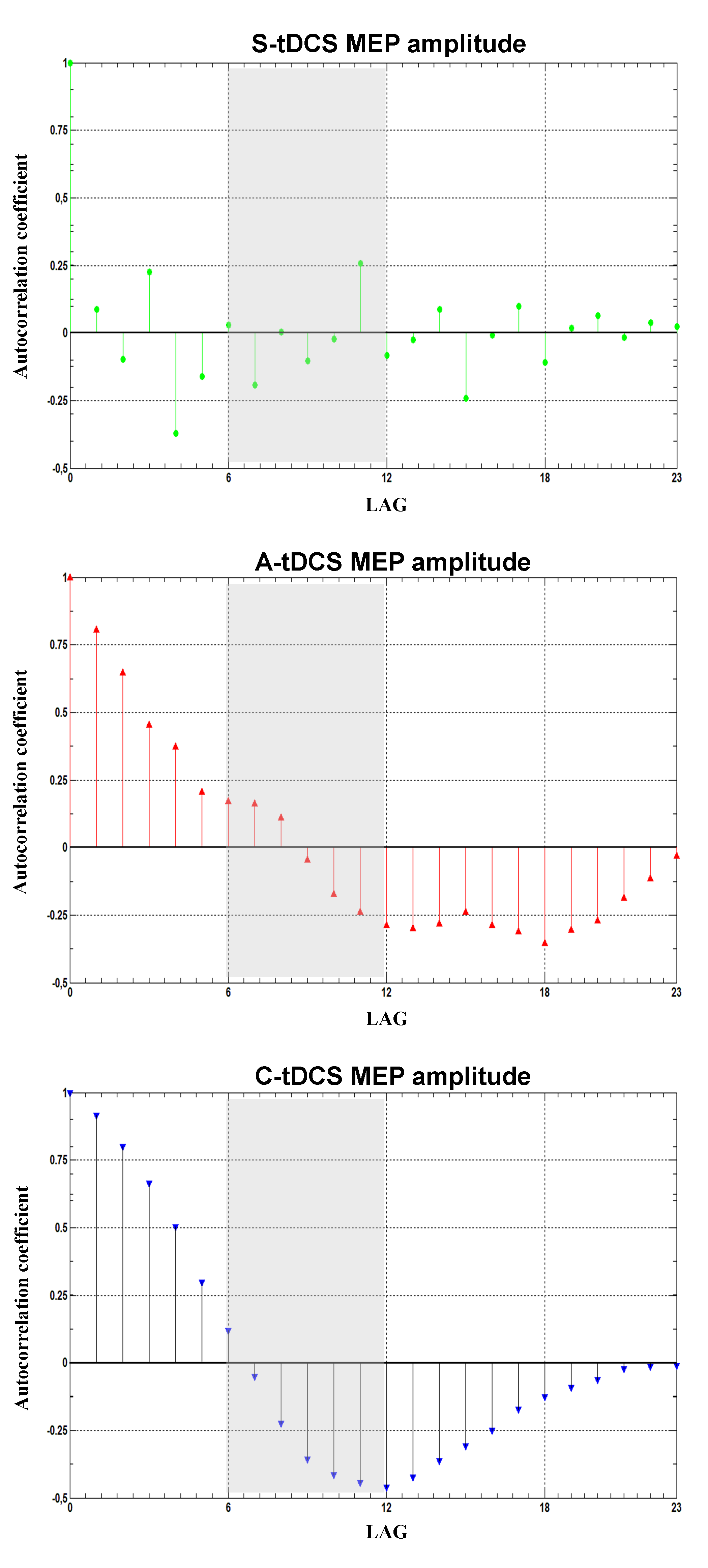

Supplement: Supplementary file 1 [file Presentation1.ZIP › Supp Fig 1.TIF]

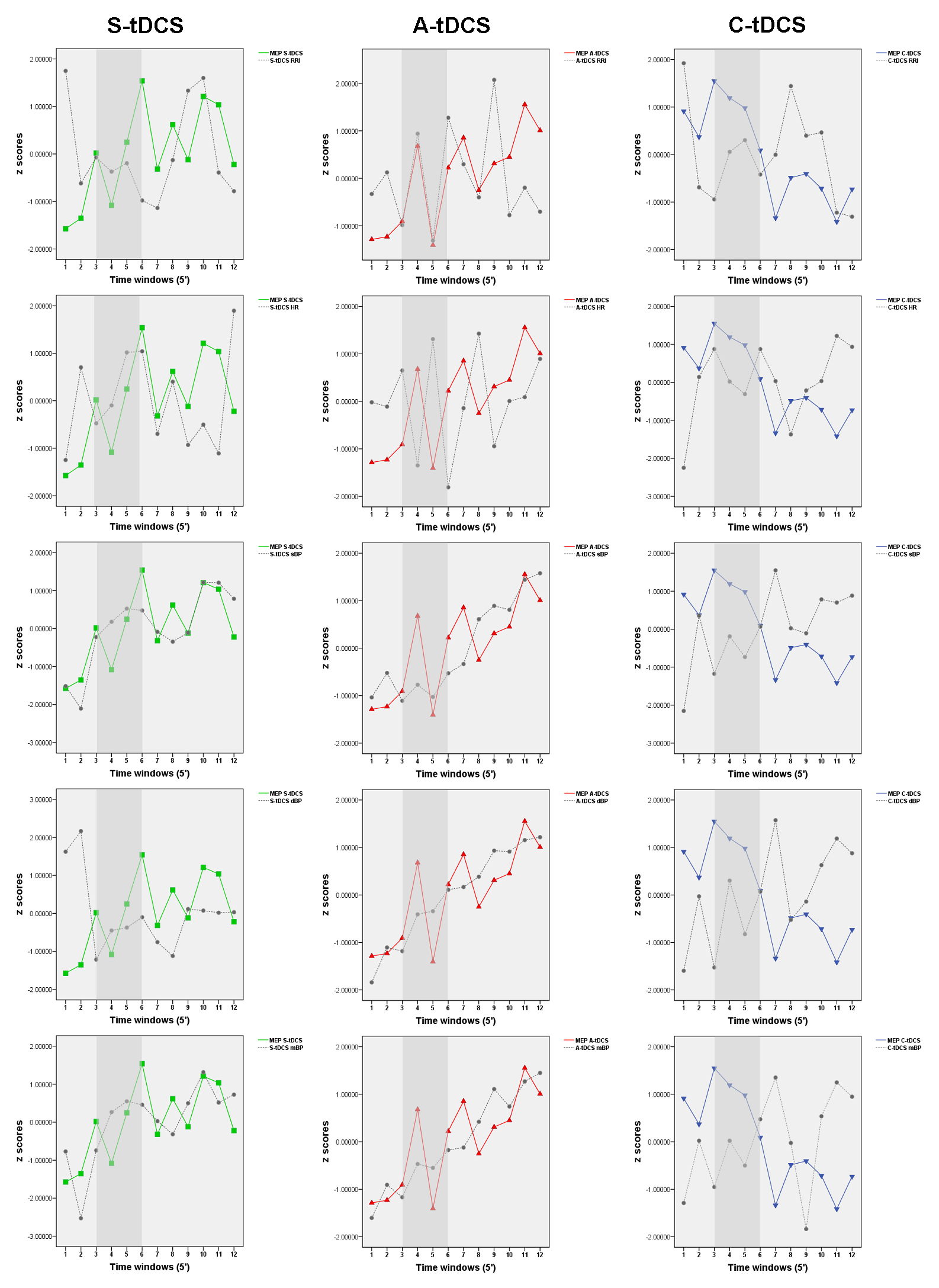

Supplement: Supplementary file 1 [file Presentation1.ZIP › Supp Fig 2.TIF]

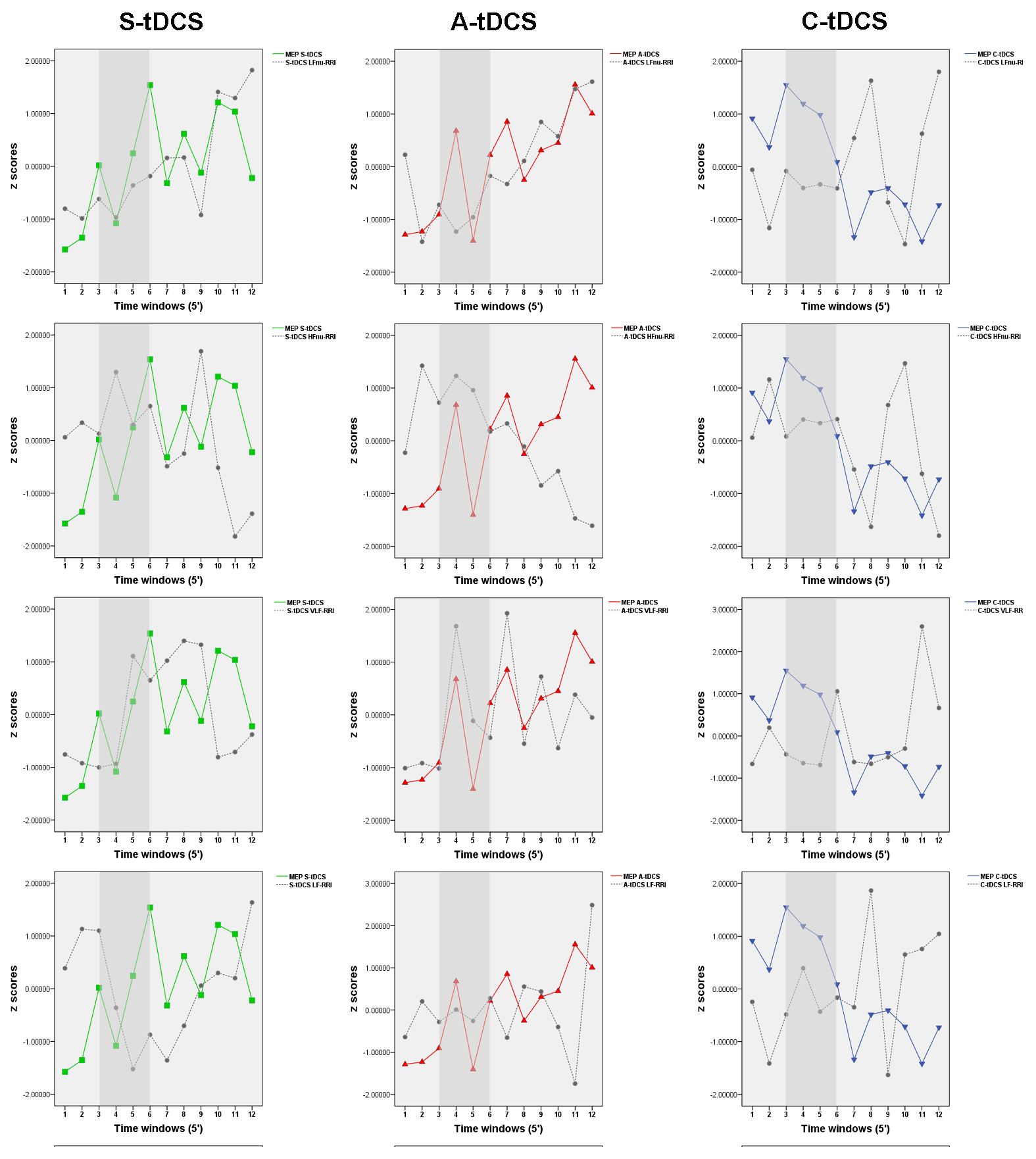

Supplement: Supplementary file 1 [file Presentation1.ZIP › Supp Fig 3.TIF]

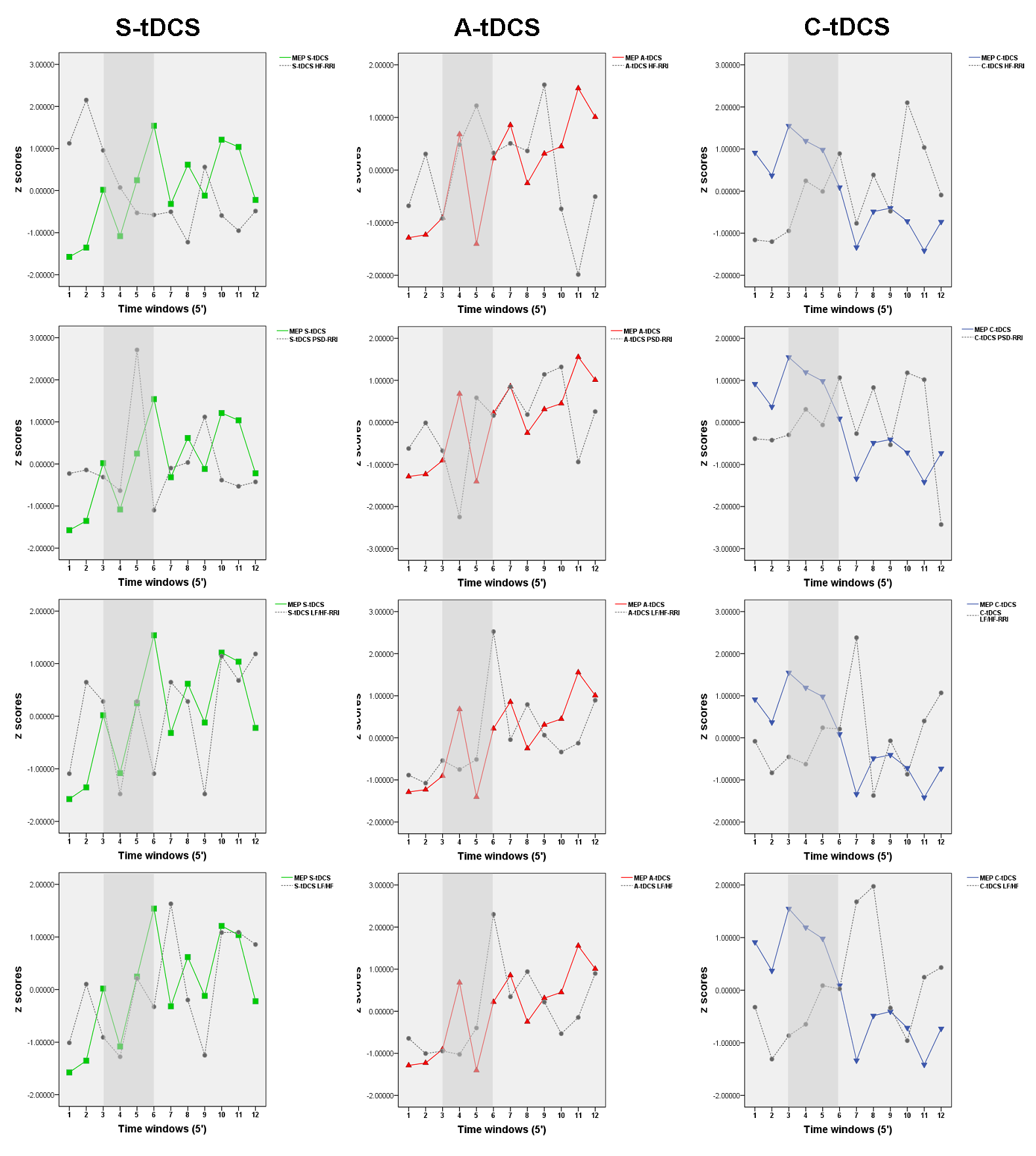

Supplement: Supplementary file 1 [file Presentation1.ZIP › Supp Fig 4.TIF]
